# Circadian cilia transcriptome in mouse brain across physiological and pathological states

**DOI:** 10.1186/s13041-024-01143-0

**Published:** 2024-09-20

**Authors:** Kiki Chen, Kousha Changizi Ashtiani, Roudabeh Vakil Monfared, Pierre Baldi, Amal Alachkar

**Affiliations:** 1 Department of Pharmaceutical Sciences, School of Pharmacy and Pharmaceutical Sciences, University of California, 356A Med Surge II, Irvine, CA 92697-4625 USA; 2 Departments of Computer Science, School of Information and Computer Sciences, University of California, Irvine, CA 92697-4625 USA; 3 Institute for Genomics and Bioinformatics, School of Information and Computer Sciences, University of California, Irvine, CA 92697 USA

**Keywords:** Cilia, Mouse, Brain, Nocturnal, Transcriptome, Circadian, Physiology

## Abstract

Primary cilia are dynamic sensory organelles that continuously undergo structural modifications in response to environmental and cellular signals, many of which exhibit rhythmic patterns. Building on our previous findings of rhythmic cilia-related gene expression in diurnal primates (baboon), this study extends the investigation to the nocturnal mouse brain to identify circadian patterns of cilia gene expression across brain regions. We used computational techniques and transcriptomic data from four publicly available databases, to examine the circadian expression of cilia-associated genes within six brain areas: brainstem, cerebellum, hippocampus, hypothalamus, striatum, and suprachiasmatic nucleus. Our analysis reveals that a substantial proportion of cilia transcripts exhibit circadian rhythmicity across the examined regions, with notable overrepresentation in the striatum, hippocampus, and cerebellum. We also demonstrate region-specific variations in the abundance and timing of circadian cilia genes’ peaks, indicating an adaptation to the distinct physiological roles of each brain region. Additionally, we show that the rhythmic patterns of cilia transcripts are shifted under various physiological and pathological conditions, including modulation of the dopamine system, high-fat diet, and epileptic conditions, indicating the adaptable nature of cilia transcripts’ oscillation. While limited to a few mouse brain regions, our study provides initial insights into the distinct circadian patterns of cilia transcripts and highlights the need for future research to expand the mapping across wider brain areas to fully understand the role of cilia’s spatiotemporal dynamics in brain functions.

**Supplementary Information:**

The online version contains supplementary material available at 10.1186/s13041-024-01143-0.

## Introduction

Primary cilia, once considered vestigial structures, are now recognized as key players across a wide range of biological processes. Extended from the majority of brain cell types, cilia serve as essential sensory and signaling hubs integral to cellular homeostasis [1, 2], development [3, 4], and signal transduction [5, 6], facilitating cell-to-cell communication and various processes critical for brain development and maturation [5, 7–11]. Functioning as cellular “antennas,” cilia detect and transduce a wide range of extracellular and environmental stimuli, including light, odorants, mechanical stimuli, and chemical signals such as neurotransmitters, hormones, and growth factors [12–15]. This signal transduction role is facilitated by receptors located on cilia membranes, including ion channels, receptor tyrosine kinases (RTKs), and G-protein-coupled receptors (GPCRs) [16–20].

Primary cilia are not static, but rather, highly dynamic structures that undergo continuous architectural changes to tailor their sensory capacities in response to various environmental cues and cellular signaling events [11, 13, 14, 21–24]. This dynamism is integral to how cilia exert their versatile functions in cellular signaling and homeostasis. For example, mechanical forces and intracellular signaling pathways mediated by cyclic AMP (cAMP) or calcium can modulate their length [25–28]. Several neurotransmitters such as dopamine [29–31], serotonin [32, 33], melanocortin [34, 35], and melanin concentrating hormone [36, 37] have been shown to modulate primary cilia structure and signaling. We have previously demonstrated that the expressions of genes coding structural and functional components of cilia exhibit change over human life-span [38]. This suggests that cilia play distinct roles in the development- and age-related functioning of brain circuits and may be involved in the pathophysiological mechanisms underlying neurodevelopmental and age-related neuropsychological disorders. Our and other studies have also established the connection between primary cilia to the pathobiology of major psychiatric disorders, demonstrating differential expression of cilia-associated genes in conditions like schizophrenia, autism spectrum disorder (ASD), bipolar disorder, depression, and Alzheimer’s disease [39–43].

The dynamic nature of cilia, along with the oscillatory stimuli they sense and the circadian patterns of physiological processes influenced by cilia signaling, such as sleep-wake cycles [44, 45], feeding [19, 34, 46], and energy balance [47–51], temperature [23, 52, 53] strongly suggests that genes responsible for cilia’s structural and functional components may exhibit rhythmic expression patterns [19]. We have provided evidence for the rhythmic patterns of cilia-associated gene expression across primate brain regions (baboon) [54]. Building upon these findings in the diurnal species, we aimed here to extend our research to a nocturnal species, the mouse. We were particularly motivated by our extensive analysis of cilia morphology across mouse brain regions, which uncovered 24-hour fluctuations in cilia length and orientation, exhibiting variations across the light and dark phases [55]. Therefore, we utilized publicly available transcriptomic data from four datasets to investigate the circadian patterns of cilia gene expression within six distinct mouse brain regions: the brainstem (BS), cerebellum (CER), ventral hippocampus (HIPP), hypothalamus (HYP), suprachiasmatic nucleus (SCN), and ventral striatum (STR). Additionally, we evaluated the impacts of various physiological and pathological conditions on the circadian patterns of cilia-associated genes in the HIPP, suprachiasmatic nucleus, and STR. These regions were specifically selected for the availability of their full circadian transcriptomics data.

## Methods and experimental designs

### Cilia genes’ list

We used a list of genes identified and verified as cilia-associated genes from the CiliaCarta database (Table S1) [56]. These genes are referred to as ‘cilia genes’. Since identifying new cilia genes is an ongoing process, leading to continuous updates in cilia gene databases, our current list of 956 cilia genes may be revised in the future.

### Circadian cilia genes data

We analyzed the circadian expression patterns of cilia-associated genes across six mouse brain regions, utilizing time series data extracted from comprehensive transcriptomic studies. Detailed descriptions of the protocols, ethics, sample collection, and methodologies can be found in the respective original publications [57–60]. These regions included the BS [57], CER [57], HIPP [58], HYP [57], SCN [59], and STR [60]. The selection of these databases was made based on their public availability and inclusion in CircadiOmics [61–64]. Out of the 956 cilia genes, different datasets included different numbers: BS, CER, and HYP included 934 cilia genes each; the hippocampus dataset included 504 cilia genes; the SCN dataset included 806 cilia genes; and the STR dataset included 794 cilia genes (Table S2). Additionally, we examined the effects of different physiological and pathological conditions on the circadian patterns of cilia gene expression within the STR, HIPP, and SCN. For the striatum, we analyzed transcriptomic data from wild-type control (WT-ctrl) mice, WT mice treated with cocaine (WT-COC), and dopamine D2 receptor knockout (D2R-KO) mice. In the hippocampus, we compared control mice to those subjected to a pilocarpine-induced epilepsy model (Epilep). For the SCN, we focused on the circadian cilia transcriptomic patterns in mice fed a balanced diet versus those on a high-fat diet (HFD).

### Circadian analysis

We analyzed the circadian patterns of cilia genes within transcriptomic time series datasets using BIO_ CYCLE, a deep-learning-based software developed to analyze periodicity in time series data [65, 66]. BIO_CYCLE estimates amplitude and phase in addition to calculating the p-value. To classify a gene’s transcript as circadian, we used a threshold of *p* = 0.05. BIO_CYCLE is accessible at the CircadiOmics Web portal: http://circadiomics.igb.uci.edu [61–64].

### Comparative analysis and functional classification of circadian cilia transcripts

A Fisher exact test was utilized to compare the ratios of circadian cilia transcripts across the six brain regions with the circadian transcriptome background of each respective region, using a p-value threshold of 0.05. This test also provided the odds ratios (OR), which quantify the likelihood that cilia genes exhibit circadian rhythms compared to non-cilia genes in the background transcriptome. Due to the data being extracted from different studies, we did not perform an overlap test of circadian cilia genes between the six regions.

Circadian cilia transcripts were classified into four distinct temporal phases, each covering a six-hour interval, representing peak expression times: first quarter-phase (ZT0-ZT5), second quarter-phase (ZT6-ZT11), third quarter-phase (ZT12-ZT17), and fourth quarter-phase (ZT18-ZT23).

The percentage of cilia transcripts peaking in each quarter-phase was then calculated.

To identify overlaps of rhythmic cilia genes among various brain regions, we performed an intersection analysis from both gene and region perspectives. Similarly, we identified overlaps of rhythmic cilia transcripts across different physiological and pathological conditions within the same brain region. Circadian cilia transcripts were further classified based on their association with cilia’s primary structural and functional components, such as the centrosome, basal body, axoneme, kinesin, dynein, Intraflagellar transport particles A and B (IFTA and IFTB), transition zone, BBSome, microtubule associated complex (MAC), and the ciliary membrane. This classification was performed using Gene Ontology (GO) annotations [67, 68]. The proportion of cilia transcripts exhibiting circadian rhythms within each sub-structure was calculated.

### Reactome enrichment

We used the PANTHER (Protein ANalysis THrough Evolutionary Relationships) tool for Reactome enrichment analysis to identify the specific pathways associated with the circadian regulation of cilia genes [69–71]. The analysis type was the PANTHER Overrepresentation Test (Released 20240226), using Reactome version 86 (released 2023-09-07). Reactome pathways analysis was performed, using the Mus musculus genome as the reference list. Pathways with false discovery rate (FDR) adjusted P value < 0.05 were considered significantly enriched [72, 73].

## Results

### Circadian oscillation of cilia transcripts in the brain is region-specific

**Fig. 1:**
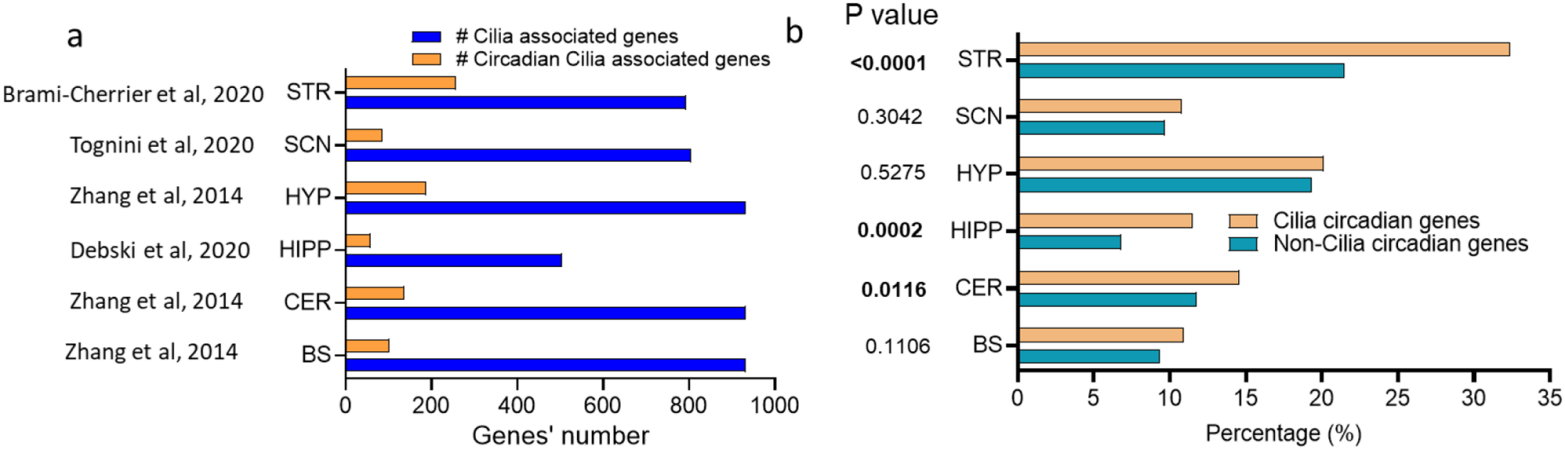
Distribution and enrichment of circadian cilia genes across six mouse brain regions. **a** Histogram showing the number of cilia cycling transcripts and total cilia transcripts in six brain regions: the brainstem (BS) [57], cerebellum (CER) [57], hippocampus (HIPP) [58], hypothalamus (HYP) [57], suprachiasmatic nucleus (SCN) [59], and striatum (STR) [60]. **b** Histogram illustrating the enrichment analysis (Fisher’s Exact Test) for circadian cilia genes across six brain regions. Significant enrichment was observed in three regions

All six examined mouse brain regions exhibited circadian cilia transcripts (Fig. 1a, Table S3), with 501 cilia genes exhibiting circadian patterns in at least one region. The STR exhibited the highest number of circadian cilia genes (257) followed by HYP (188), CER (136), BS (102), and SCN (87) accounting for 32.4%, 20.1%, 14.6%, 10.9%, and 10.8% of their respective total cilia genes (Fig. 1a). The HIPP presented the fewest circadian genes (58 cycling cilia-associated genes), which accounts for 11.5% of its cilia genes. Notably, the Syne1 gene contributed 12 isoforms of these genes. Fisher’s exact test showed that circadian rhythmicity in cilia genes was significantly overrepresented compared to background genes in the STR (OR 1.75), HIPP (OR 1.78), and CER (OR 1.28) (*P* < 0.05) (Fig. 1b).

**Fig. 2:**
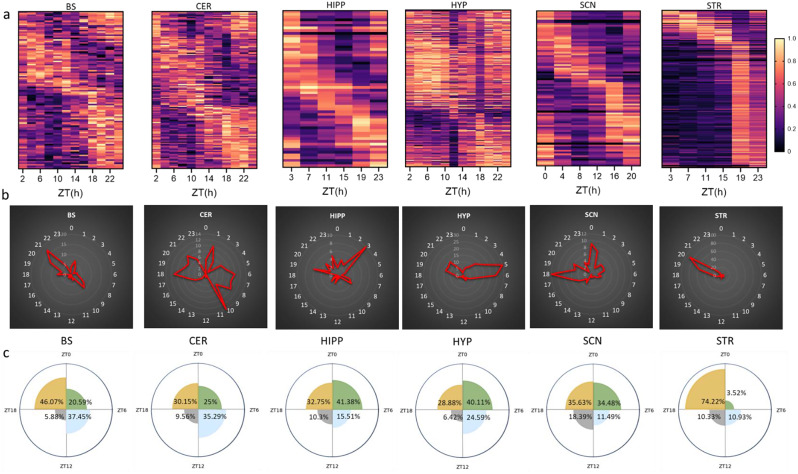
Region-specific expression patterns of circadian cilia genes. **a** Heatmap visualization of the 24-hour oscillation patterns of circadian cilia genes across six mouse brain regions (*P* < 0.05). Gene expressions are normalized between 0 and 1, yellow (1) indicates peak expression and dark navy (0) indicates a trough of expression. **b** Radial plot showing the peak expression phases of circadian cilia genes in each of the six brain regions, with phases (hours) marked along the circumference and the number of gene expression peaks represented along the radius. **c **Rose diagram illustrating the distribution of circadian cilia genes across four quarter-phases, represented as percentages

We categorized the peaks’ distribution of circadian cilia transcripts into four 6-hour phases (quarter-phases). Our analysis revealed distinct temporal patterns of circadian cilia gene expression across the six mouse brain regions. In the BS, CER, and HYP, circadian cilia genes demonstrated peak expressions spanning multiple phases. In the BS, a predominant majority peaked during the fourth phase (46%), followed by the second (37.45%) and first (20.59%) phases, with peaks at ZT1, ZT9, and ZT21. Similarly, in the CER, the distribution was across the first (25%), second (35.29%), and fourth (30.15%) phases, with a sharp peak at ZT10, and less profound peaks at ZT1, ZT6, ZT8 and ZT18 (Fig. 2a-c). In the HYP, a predominant majority peaked during the first phase (40.11%), followed by the fourth (28.88%) and second (24.59%) phases, with sharp peaks at ZT5, ZT6, and less profound peaks at ZT20 and ZT21. The HIPP and SCN displayed comparable circadian patterns in cilia gene expression, predominantly in the first and fourth quarter-phases. The HIPP showed a major distribution of circadian cilia genes peaking in the first (41.38%) and fourth (32.75%) quarter-phases, with sharp peaks at ZT3, and less profound at ZT8, ZT19, and ZT23. The SCN presented an even distribution in the first (34.48%) and fourth (35.63%) phases, with the bulk of circadian cilia genes peaking at ZT0 and ZT18 (Fig. 2a-c). A narrow distribution of circadian cilia gene patterns was observed in the STR, with most circadian cilia genes peaking in the fourth quarter-phase (74.22%), sharply concentrated around ZT20. Minor peaks were observed in the second and third phases, each around 10% (Fig. 2a-c).

### Structural and functional organization of circadian cilia genes in the BS, CER, and HYP

**Fig. 3:**
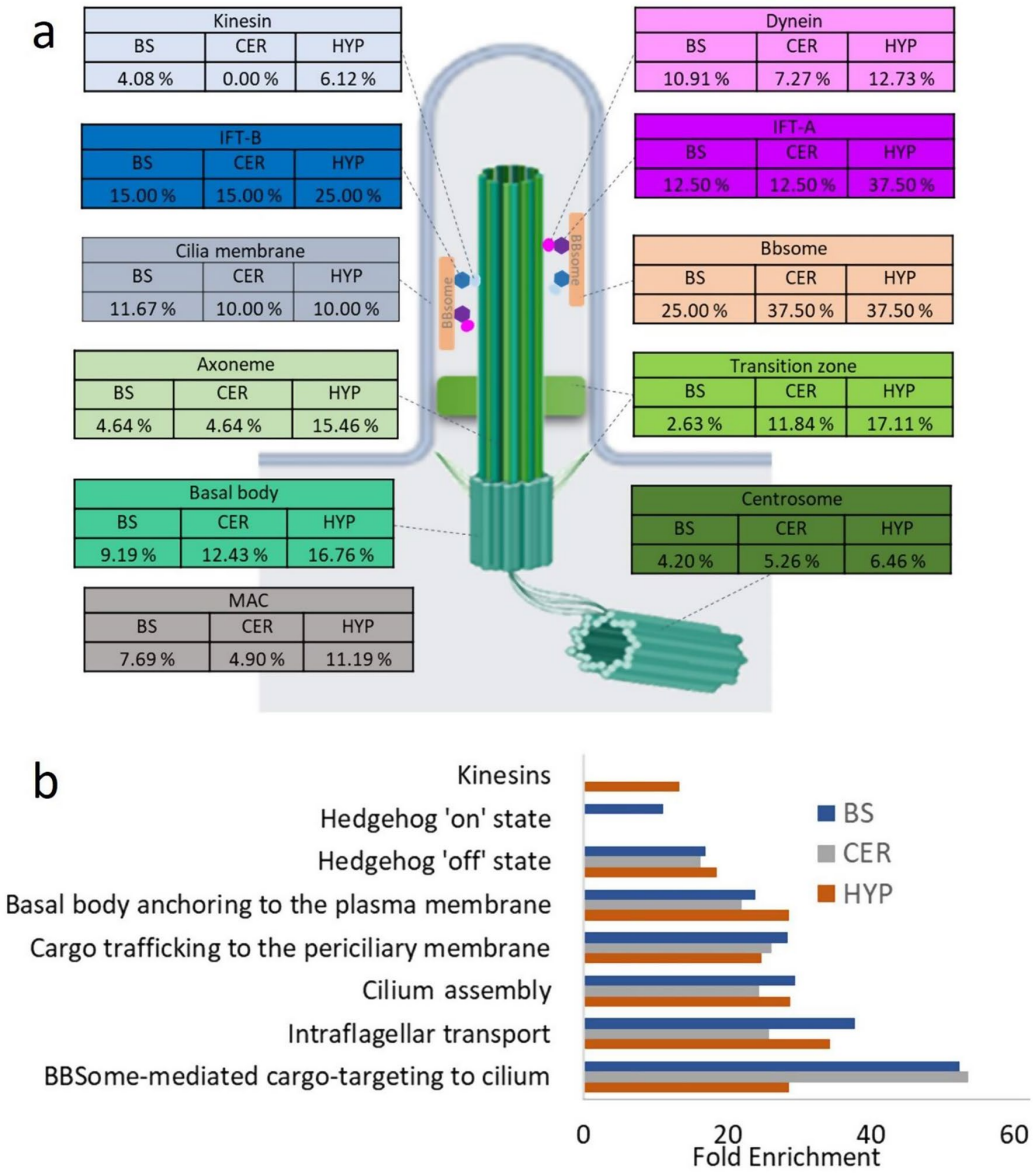
Structural and functional organization of circadian cilia genes in the BS, CER, and HYP **a** Schematic of cilia structure showing the percentage of circadian genes within each sub-structural compartment in the BS, CER, and HYP. **b** The Reactome pathway enrichment analysis of circadian cilia genes in the BS, CER, and HYP. Statistically significant pathways are represented as Fold enrichment (FDR < 0.05)

We examined the distribution of circadian cilia transcripts across the main structural and functional components of cilia in the BS, CER, and HYP, as these regions were analyzed from the same dataset, enabling direct comparison. The HYP generally had a higher proportion of circadian genes in most cilia substructures compared to BS and CER (Fig. 3a, Table S4). Notably, the BBSome showed the highest circadian gene percentage in the CER and HYP (37.50%), compared to 25.00% in the BS. Intraflagellar transport particles A and B (IFT-A and IFT-B) showed notable circadian gene presence in the HYP at 37.50% and 25.00%, respectively, compared to 12.50% and 15.00% for IFT-A and IFT-B in both the BS and CER. The percentage of circadian genes in the ciliary membrane were relatively consistent across the three regions, around 10%. The kinesin complex showed low percentages of circadian genes in the BS and HYP and no circadian genes in CER (Fig. 3a, Table S4).

To identify the cilia pathways involving circadian cilia genes, Reactome pathways enrichment analysis was carried out. BBSome-mediated cargo was highly enriched in the CER (53.75-fold enrichment (FE)) and HYP (52.49), compared to the BS (28.63). IFT showed notable enrichment in the BS and HYP, with a slightly lower enrichment fold in the CER (Fig. 3b). Cilium assembly and basal body anchoring to the plasma membrane were consistently enriched across the BS, CER, and HYP. While the Hedgehog ‘off’ state pathway showed relatively similar enrichment across the three regions, the hedgehog ‘on’ state was only enriched in the BS The kinesin pathway also showed an enrichment of in HYP with no significant enrichment in the other regions (Fig. 3b).

### Changes in circadian patterns of cilia genes under various physiological and pathological conditions

We analyzed the changes in circadian patterns of cilia genes under various physiological and pathological conditions in three of the six studied regions, based on the availability of datasets. We examined the effects of cocaine-induced activation of dopamine system and selective D2R deletion in medium striatal neurons (MSNs) on circadian cilia gene patterns. We also examined the effects of HFD and epileptic status on the circadian patterns of cilia genes in the SCN and the ventral HIPP respectively.

#### Striatum

**Fig. 4:**
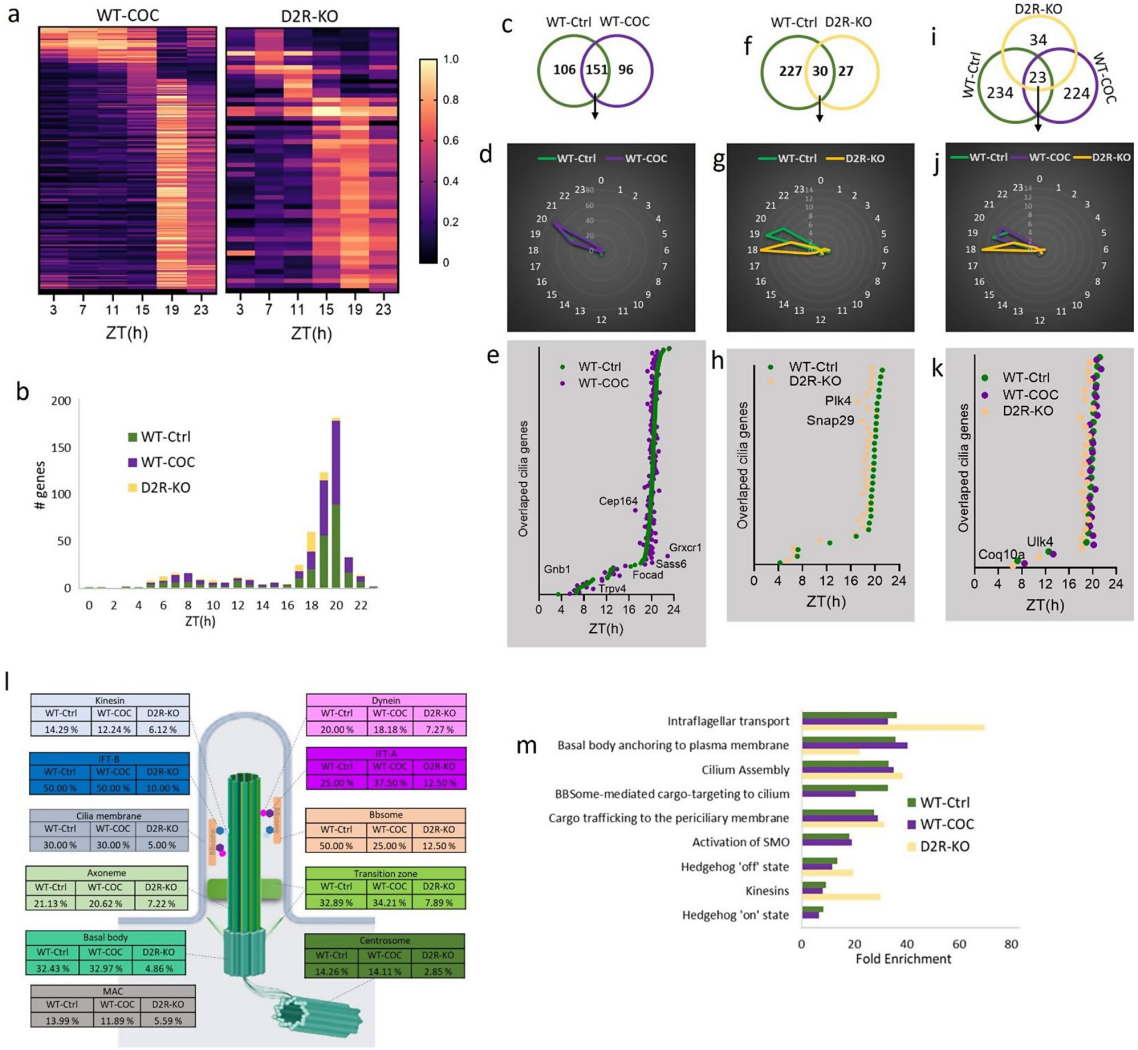
Structural and functional organization of circadian cilia genes in the striatum under cocaine and D2R deletion conditions. **a** Heatmap visualization of the 24-hour oscillation patterns of circadian cilia genes in the STR of WT-COC, and D2R-KO mice (*P* < 0.05). Gene expressions are normalized between 0 and 1, yellow (1) indicates peak expression and dark navy (0) indicates a trough of expression. **b** Histogram showing the distribution of peak expression phases for cilia associated genes in the STR of WT-ctrl, WT-COC, and D2R-KO mice, with the x-axis indicating the hour of the day, labeled as ZT. **c** Venn diagram displaying the overlap of cyclic transcripts in the STR between control and cocaine-treated conditions. **d** Radial plot illustrating peak expression phases of circadian cilia genes in the STR of WT-Ctrl vs. WT-COC, with phase hours marked on the circumference and gene peak counts on the radial axis. **e** phase plot showing the peak expression times (ZT) of overlapping cilia genes in WT-Ctrl and WT-COC mice over a 24-hour period. Genes with peak time shifts greater than 2 h are labeled. **f** venn diagram displaying the overlap of cyclic transcripts in the STR between WT-Ctrl and D2R-KO conditions. **g** Radial plot illustrating peak expression phases of circadian cilia genes in the STR of WT-Ctrl and D2R-KO, with phase hours marked on the circumference and gene peak counts on the radial axis. **h** Phase plot showing the peak expression times (ZT) of overlapping cilia genes in WT-Ctrl and D2R-KO mice over a 24-hour period. Genes with peak time shifts greater than 2 h are labeled. **i** Venn diagram displaying the overlap of cyclic transcripts in the STR between WT-Ctrl, WT-COC, and D2R-KO conditions. **j** Radial plot illustrating peak expression phases of circadian cilia genes in the STR of WT-Ctrl, WT-COC, and D2R-KO, with phase hours marked on the outer circle and gene peak counts on the radial axis. **k** Phase plot showing the peak expression times (ZT) of overlapping cilia genes in WT-Ctrl, WT-COC, and D2R-KO mice over a 24-hour period. Genes with peak time shifts greater than 2 h are labeled. **l** Schematic of cilia structure displaying the percentage of circadian genes within each sub-structural compartment in the STR of WT-Ctrl, WT-COC, and D2R-KO. **m** The Reactome pathway enrichment analysis of circadian cilia genes in the STR of WT-Ctrl, WT-COC, and D2R-KO. Statistically significant pathways are represented as Fold enrichment (FDR < 0.05)

Under cocaine treatment, 247 cilia cycling genes were identified in the STR, resembling the distribution pattern observed in control mice (Fig. 4a, b, Table S3), with 151 of these genes overlapping between both conditions (Fig. 4c, Table S3). While these overlapping genes maintained a similar distribution and sharply peaked at ZT20 (Fig. 4d), some showed shifts in their peak times: seven genes exhibited earlier peak times by more than one hour, with one gene peaking two hours earlier (Cep164), and 16 genes showed delayed peak times by more than one hour, with five genes peaking over two hours later (Sass6, Focad, Gnb1, Trpv4, Grxcr1) in the STR of cocaine-treated mice (Fig. 4e).

D2R deletion in the STR reduced the number of circadian cilia genes to 57, with only 30 genes overlapping between D2R-KO and WT mice (Fig. 4a.f). Notably, all except one of these overlapping genes shifted to peak earlier, around ZT18-19. Of these, 18 genes peaked one hour earlier, and 2 of them (Plk4and SNAP29) peaked two hours earlier in D2R-KO compared to WT mice (Fig. 4f-h).

Only 23 genes were found to overlap across the WT-Ctrl, WT-COC, and D2R-KO conditions, with these shared genes exhibiting a comparable distribution across all three conditions (Fig. 4i-k). Our analysis of circadian cilia gene expression across the STR’s main cilia structural and functional components under WT-Ctrl, WT-COC, and D2R-KO conditions revealed roughly similar proportions of circadian genes within the majority of cilia substructure for WT-Ctrl and WT-COC, but lower proportions for D2R-KO. The centrosome components showed the highest number of circadian cilia genes in the WT-Ctrl condition, with 95 out of 666 centrosome genes being circadian, accounting for 14.26% of the total centrosome genes. This proportion remained similar in the WT-COC condition, with 94 circadian genes (14.11%). However, in the D2R-KO condition, the number of circadian genes decreased significantly to 19, accounting for only 2.84% of the total centrosome genes (Fig. 4l, Table S4). The basal body contained the second highest number of circadian genes in the WT-Ctrl (60 genes) and WT-COC (61 genes), accounting for 32.43% and 32.97% of the basal body genes, respectively. In contrast, only 9 basal body genes (4.86%) were circadian in the D2R-KO condition. Notably, 50% of the BBSome components exhibited circadian patterns in WT-Control, compared to 25% in WT-COC and 12.5% in D2R-KO conditions (Fig. 4l, Table S4). The axoneme had 21.13% of its genes as circadian in WT-Ctrl, 20.62% in WT-COC, and 7.22% in D2R-KO. The ciliary transition zone showed 32.89% circadian genes in WT-Ctrl, 34.21% in WT-COC, and 7.89% in D2R-KO. The ciliary membrane maintained 30% circadian genes in both WT-Ctrl and WT-COC, dropping to 5% in D2R-KO. IFT-B remained at 50% in both WT-Ctrl and WT-COC, but fell to 10% in D2R-KO, while IFT-A increased from 25% in WT-Ctrl to 37.50% in WT-COC and decreased to 12.50% in D2R-KO. The dynein complex had 20% circadian genes in WT-Ctrl, 18.18% in WT-COC, and 7.27% in D2R-KO, whereas the kinesin complex showed 14.29% in WT-Ctrl, 12.24% in WT-COC, and 6.12% in D2R-KO (Fig. 4l, Table S4).

The top enriched Reactome pathways with FDR value lower than 0.05 in the WT-Control condition included IFT, basal body anchoring to the plasma membrane, cilium assembly, BBSome-mediated cargo targeting to the cilium, cargo trafficking to the periciliary membrane, activation of SMO, hedgehog ‘off’ state, kinesins, and hedgehog ‘on’ state (Fig. 4m). Similar pathways with parallel enrichment folds were observed in the WT-COC condition. In the D2R-KO condition, there was an increase in enrichment fold of IFT and kinesins pathways, whereas the enrichment dramatically decreased for BBSome-mediated cargo targeting to the cilium, activation of SMO, and hedgehog ‘on’ state (Fig. 4m).

#### Hippocampus

**Fig. 5:**
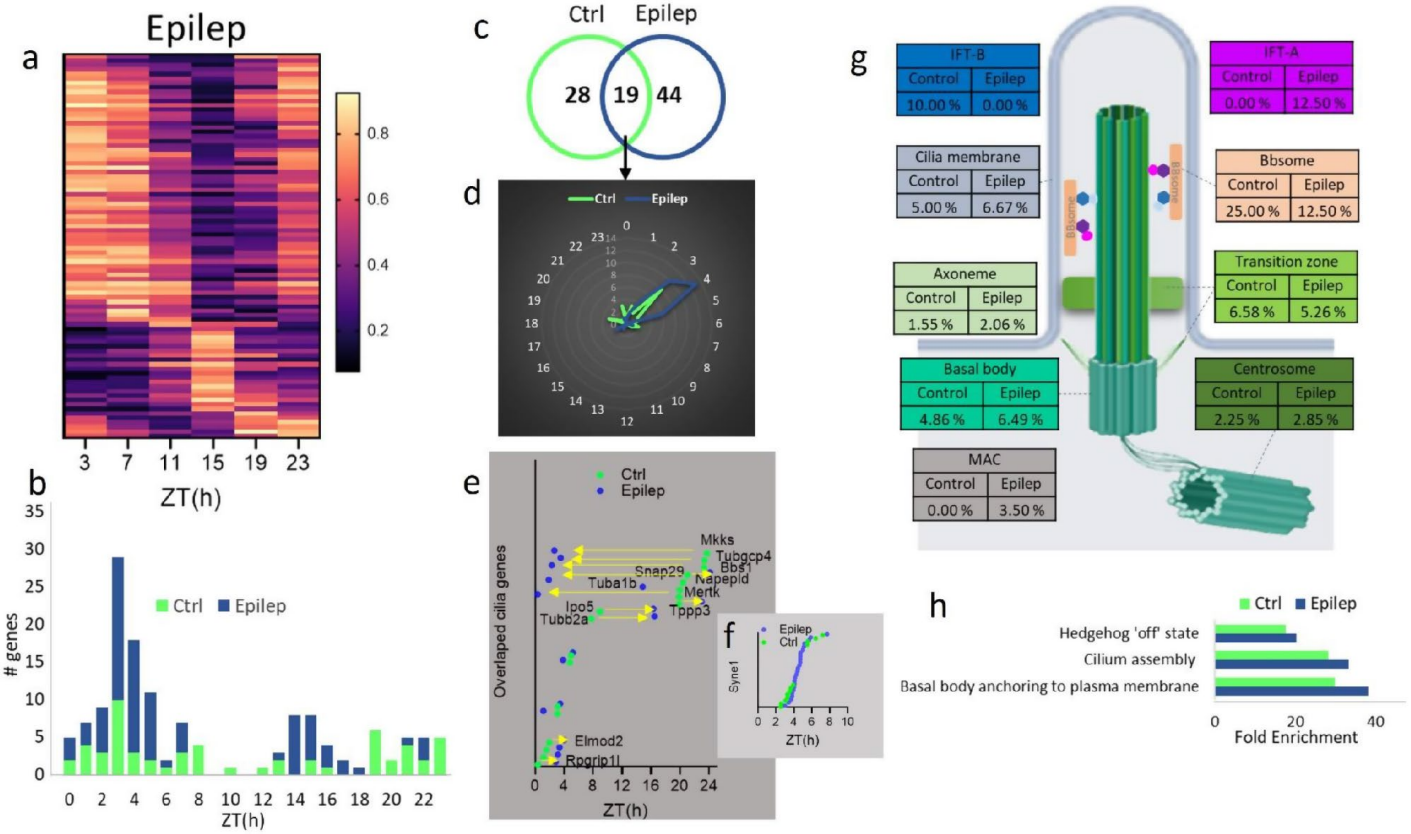
Structural and functional organization of circadian cilia genes in the hippocampus (HIPP) under epileptic condition **a** Heatmap visualization of the 24-hour oscillation patterns of circadian cilia genes in the HIPP of epileptic mice (*P* < 0.05). Gene expressions are normalized between 0 and 1, yellow (1) indicates peak expression and dark navy (0) indicates a trough of expression. **b** Histogram showing the distribution of peak expression phases for cilia associated genes in the HIPP of control and epileptic mice, with the x-axis indicating the hour of the day, labeled as ZT. **c** Venn diagram displaying the overlap of cyclic transcripts in the HIPP between control (Ctrl) and epileptic (epilep) conditions. **d** Radial plot illustrating peak expression phases of circadian cilia genes in the HIPP under control and epileptic conditions, with phase hours marked on the outer circle and gene peak counts on the radial axis. **e** Phase plot showing the peak expression times (ZT) of overlapping cilia genes in the HIPP of control and epileptic mice over a 24-hour period. Genes with peak time shifts greater than 2 h are labeled and indicated with arrows showing the direction of the shift from Ctrl to epileptic condition. **f** Phase plot showing the peak expression times (ZT) of Syne1 isoforms in the HIPP of control and epileptic mice over a 24-hour period. **g** Schematic of cilia structure showing the percentage of circadian genes within each sub-structural compartment of the HIPP of control and epileptic conditions. **h** The Reactome pathway enrichment analysis of circadian cilia genes in the HIPP of control and epileptic mice. Statistically significant pathways are represented as Fold enrichment (FDR < 0.05)

In the HIPP of epileptic mice, 86 cilia genes displayed circadian patterns, including 24 isoforms of the gene Syne1, with their distribution shifting more towards the first quarter-phase (Fig. 5a, b, Table S3). Of these genes, only 18 genes and the Syne1 isofoms were shared between the two conditions (Fig. 5c), with 7 exhibiting earlier peaks and 8 exhibiting delayed peaks in the epileptic state (Fig. 5d, e). Since the number of Syne1 isoforms is different between the control (12) and epileptic (24) conditions, we included a separate graph showing the peaking times of Syne1 isoforms, which varied widely but were parallel in both Control and epileptic conditions (Fig. 5f). Notable variations were observed in the distribution of circadian genes across different cilia substructures between the epileptic and control HIPP groups (Fig. 5g). Only 2.25% and 2.85% of the centrosome component genes were circadian in the HIPP of control and epileptic mice, respectively. While the circadian components of the axoneme and basal body increased from 1.55% and 4.86% in the control group to higher percentages in the epileptic group, the proportion of the BBSome circadian genes decreased from 25 to 12.5% of the total BBSome genes. No circadian genes were found in kinesins or dynein in either condition, while circadian genes of IFT-A were absent in the epileptic group and circadian genes of IFT-B were absent in the control group (Fig. 5f, Table S4).

The top enriched Reactome pathways with FDR value lower than 0.05 in the HIPP of control mice included basal body anchoring to the plasma membrane, cilium assembly, and the hedgehog ‘off’ state. Similar pathways were enriched in the Epilep condition, but with higher fold enrichment score (Fig. 5h).

#### SCN

**Fig. 6:**
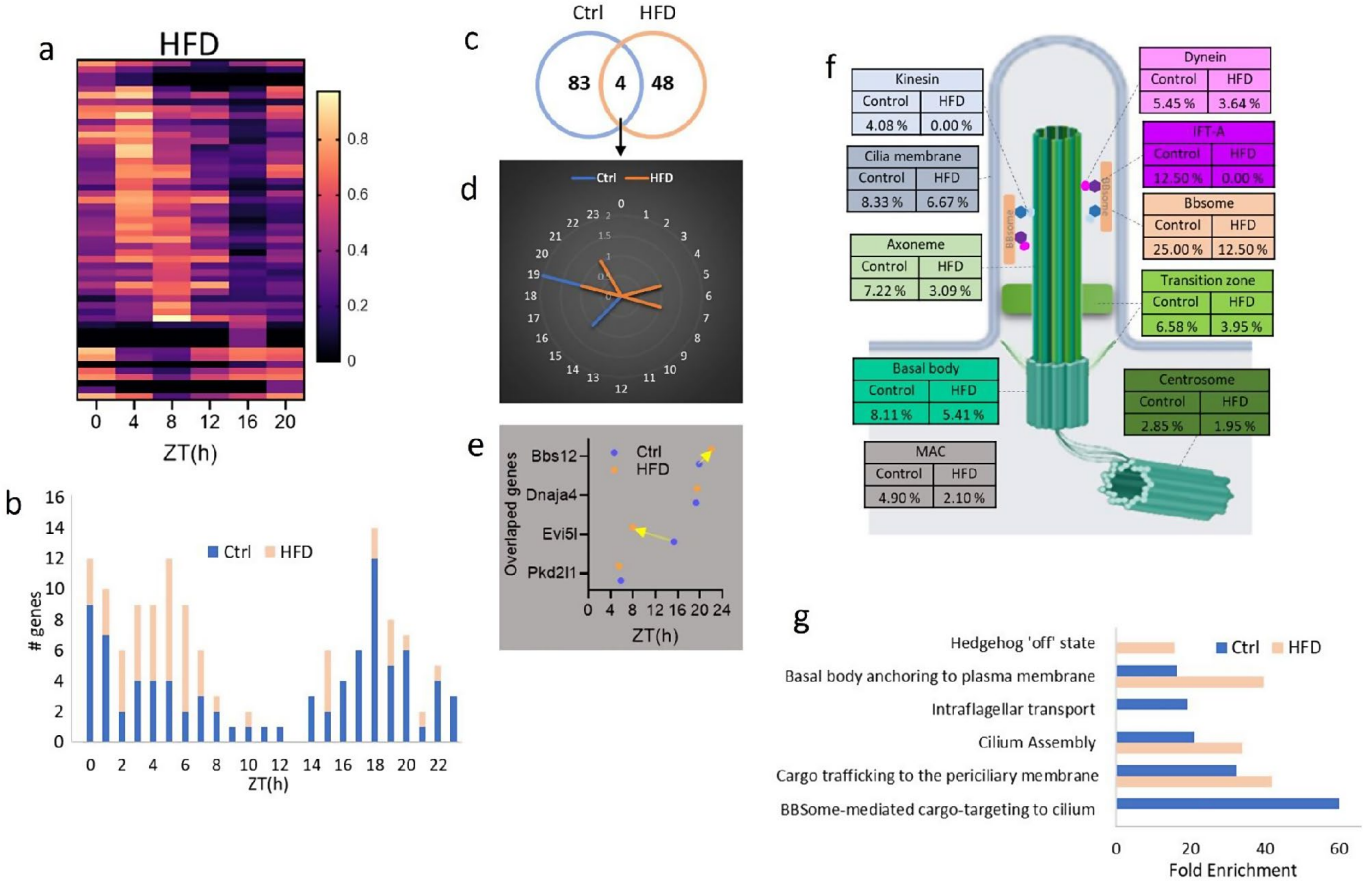
Patterns of circadian cilia transcriptomics in the SCN under HFD condition **a** Heatmap visualization of the 24-hour oscillation patterns of circadian cilia genes in the SCN of HFD mice (*P* < 0.05). Gene expressions are normalized between 0 and 1, yellow (1) indicates peak expression and dark navy (0) indicates a trough of expression. **b** Histogram showing the distribution of peak expression phases for cilia genes in the SCN of control and HFD mice, with the x-axis indicating the hour of the day, labeled as ZT. **c** Venn diagram displaying the overlap of cyclic transcripts in the SCN between control Ctrl and HFD conditions. **d** Radial plot illustrating peak expression phases of circadian cilia genes in the SCN under control and HFD conditions, with phase hours marked on the circumference and gene peak counts on the radial axis. **e** Phase plot showing the peak expression times (ZT) of overlapping cilia genes in the SCN of control and HFD mice over a 24-hour period. Genes with peak time shifts greater than 2 h are labeled and indicated with arrows showing the direction of the shift from Ctrl to epileptic condition. **f** Schematic of cilia structure showing the percentage of circadian genes within each sub-structural compartment of the SCN of control and HFD conditions. **g** The Reactome pathway enrichment analysis of circadian cilia genes in the SCN of control and HFD mice. Statistically significant pathways are represented as Fold enrichment (FDR < 0.05)

In the SCN of HFD mice, the number of circadian cilia genes decreased to 52 (Fig. 6a, b, Table S3), with only four genes—Pkd2l1, Evi5l, Dnaja4, and Bbs12—shared between the control and HFD groups (Fig. 6c-e). A notable shift from a dark-phase peak in control diet mice to a light-phase peak (ZT5-ZT7) in HFD mice was observed (Fig. 6a, b). The distribution of circadian genes within different cilia substructures varied between the control and HFD groups, showing a trend toward a decrease in the proportions of circadian genes across all cilia substructures in the SCN of HFD mice (Fig. 6f, Table S4). For instance, the proportions of kinesin and IFT-A circadian genes decreased from 4.08% and 12.50% respectively in control mice to 0% in HFD mice (Fig. 6f).

The top enriched Reactome pathways in both the control and HFD groups included cargo trafficking to the periciliary membrane, cilium assembly, and basal body anchoring to the plasma membrane (Fig. 6g). However, IFT and BBSome-mediated cargo targeting to cilium were enriched only in the control group, while the hedgehog ‘on’ state was uniquely enriched in the HFD group (Fig. 6g).

## Discussion

In this study, we extended our investigation of circadian rhythms from diurnal primates to nocturnal mice, to assess how physiological and pathological conditions affect cilia-associated gene expression across six mouse brain regions. Our findings reveal circadian rhythmicity in approximately half of the cilia transcripts and significant overrepresention in three regions, STR, HIPP, and CER, compared to the background circadian transcriptome. Notably, these rhythmic patterns are altered by various conditions, providing further evidence for cilia adaptability to environmental changes and their potential role in the regulation of brain circadian functions.

### Circadian cilia transcripts display distinct and region-specific rhythmic patterns in the mouse brain

We analyzed the circadian patterns of cilia gene expression across six mouse brain regions (BS, CER, HIPP, HYP, SCN, and STR), chosen for the availability of comprehensive circadian transcriptomics data. Our analysis revealed region-specific circadian rhythms of cilia-associated genes, highlighting the variability in circadian gene expression across the brain regions. The STR showed the highest abundance of circadian cilia-associated genes, whereas the HIPP exhibited the lowest, resembling patterns observed in our previous diurnal baboon study [54]. The distribution of circadian cilia genes’ peaking times is interesting, as all regions showed low peaking during the third quarter-phase, which represents the first half of the dark phase. In the striatum of nocturnal mice, we observed that cilia transcripts peak distinctly at ZT20, differing from the background genes which peak at ZT4-6 and ZT16-18 [60]. This also contrasts with our findings in diurnal baboons, where cilia transcripts in the putamen, a component analogous to the mouse striatum, peak around ZT4 [54]. This observation is particularly interesting given the striatum’s crucial role in motor control and executive functions, which peak during the awake phase and diminish during sleep. The difference in peak cilia gene expression between nocturnal mice and diurnal baboons suggests crucial roles for the striatal cilia dynamicity in species-specific motor activity patterns and highlights their adaptability across species.

Circadian cilia transcripts within the mouse SCN displayed peaks across the first and fourth quarter-phases, diverging from the predominant peaking in the third and fourth quarter-phases observed in most background circadian transcripts in this region [59]. This distribution resembles patterns seen in baboons [54], with a notable distinction in mice: a pronounced peak at ZT0 (close to the dark-to-light transition) and at ZT18 (the beginning of the fourth quarter-phase). The SCN acts as the master clock of the mammalian circadian system, receiving environmental cues, primarily light, to synchronize the body’s internal clock with the 24-hour light/dark cycle. A recent study showed that cilia in a subpopulation of the SCN neurons exhibit pronounced circadian rhythmicity in abundance and length, driving oscillations of Sonic Hedgehog (Shh) signaling and clock gene expression [74]. In the HYP, circadian cilia genes peak during the first, second, and fourth quarter-phases, differing from baboon hypothalamic nuclei, which predominantly peak during the dark phase [54]. This suggests an adaptability of cilia gene expression patterns to the distinct activity cycles of each species.

The peak distribution of circadian cilia genes in the BS and CER across the first, second, third, and fourth quarter-phases reveals notable similarities. Both regions show a predominant peaking of circadian cilia genes during the second and fourth quarter-phases, with a notably low presence during the third quarter-phase. This similarity in peak distribution between the BS and CER might reflect their interconnected roles in coordinating motor functions and maintaining balance. The BS, being a crucial relay center for signals between the brain and the body, integrates sensory and motor pathways. The CER, which plays a key role in motor control, receives inputs from the BS and further processes these signals to fine-tune motor activities. Thus, the observed circadian gene expression patterns might reflect the synchronization of motor-related processes in these two regions, aligning with the nocturnal activity cycle of mice. The low expression during the third quarter-phase (representing early dark phase) suggests a period of transition in activity, while the higher peaks during the second and fourth quarter-phases (late light phase and late dark phase) indicate times of increased motor function and coordination, consistent with the active period of nocturnal mice during the dark phase and their preparation for rest during the light phase.

BBS2, which plays a role in ciliary protein trafficking, showed circadian expression across the BS, CER, and HYP, suggesting that it may play a role in synchronizing physiological processes in these regions. Notably, BBS2 has been shown to be essential for leptin signaling, which exhibits circadian rhythmicity and plays an important role in the functions of the HYP, BS, and CER [75–80].

The enrichment analysis suggests an important role of circadian regulation in cilia functionality across different brain regions. The unique enrichment of the Hedgehog ‘on’ state in the HYP supports distinct circadian signaling essential for hypothalamic functions. A previous study has demonstrated a role of the hypothalamic Hedgehog signaling in functions like feeding, which is rhythmic in nature [81].

### Circadian cilia transcripts’ patterns are altered in various physio-pathological states

Numerous physiological functions regulated by cilia are circadian in nature. We, therefore, examined how different physiological and pathological conditions impact the circadian patterns of cilia transcriptome. We first studied the effects of modulating the dopamine system, specifically through cocaine treatment to activate the system and D2R knockout for inactivation. While cocaine treatment, which increases synaptic dopamine by inhibiting its transporter, slightly reduced the number of circadian cilia transcripts, D2R deletion led to a profound decrease. Notably, cocaine treatment maintained the overall distribution pattern of circadian cilia genes, whereas D2R deletion shifted their phase distribution to earlier times. Given that cocaine leads to the activation of both D1R and D2R, while D2R deletion shifts dopamine’s effects to D1R activation alone, this raises the question of whether D1R plays a dominant role in modulating circadian gene expression in cilia, particularly that about 60% of ventral-STR MSNs express D1R, compared to 50% expressing D2R [82, 83].

D1R and D2R are among the few GPCRs known for their cilia localization in addition to their location on neuronal cell membranes [16–19]. D1R and D2R exhibit contrasting effects on cilia morphology: D1R activation promotes cilia elongation, whereas D2R activation leads to cilia shortening [31, 84–88], suggesting that cilia harboring D1R and D2R in the striatum respond differently to dopamine. Studies have demonstrated a critical role of D1R positioning on cilia for its signaling and functions in the STR [89]; disruptions in D1R positioning on cilia impair signaling in striatal neurons, and neurons lacking cilia exhibit reduced D1R-dependent signaling pathways [89]. D1R and D2R are expressed by distinct neuronal populations within the STR, the basal ganglia’s principal input region, where D1R activation enhances the direct pathway, and D2R activation suppresses the indirect pathway of the basal ganglia circuit (for review: [90, 91]). In addition to its role in motor and executive functions, the STR also plays an essential role in the brain’s clock mechanisms, influencing interval timing—the ability to judge time durations from seconds to minutes [92]. The STR’s contribution to interval timing relies on dopaminergic projections from the substantia nigra compacta (SNc), with neuron action potentials acting as pacemaker pulses that adjust the speed of the internal clock [92–95]. Changes in dopamine levels influence time perception by modulating the speed of the internal clock: increased striatal dopamine speeds up the clock, while reductions slow it down [93–95]. Thus, drugs boosting dopamine levels, like cocaine and methamphetamine, result in quicker timing judgments and shifts towards earlier timing, whereas dopamine receptor antagonists slow the clock, leading to delayed timing judgments [96–99]. Our recent study highlighted the importance of cilia in the STR by showing that cilia ablation in the mouse STR impairs time perception, inhibiting the ability to quickly adapt behavior to environmental changes [100].

Our findings demonstrate that pilocarpine-induced epilepsy profoundly increases the number of circadian cilia-associated genes in the hippocampus, with a notable rise in 24 Syne1 isoforms exhibiting circadian patterns in the epileptic state compared to 12 in the control. Syne1, which encodes multiple isoforms of Nesprin1 found in cilia rootlets, is predominantly expressed in regions critical for synaptic plasticity, including the HIPP, cerebral cortex (CTX), STR, and CER, with evidence for increased demand for SYNE1 during synaptic plasticity periods [101]. Interestingly, we found that the STR and CER at normal conditions contained only one circadian isoform of Syne1. Large-scale GWAS have identified Syne1 as a top risk locus for major psychiatric disorders including schizophrenia, bipolar disorder, major depression, ADHD, and autism spectrum disorder, with particularly strong associations with bipolar disorder, second only to ANK3 [102, 103]. Beyond psychiatric implications, Syne1 has been linked to a range of genetic disorders, such as autosomal recessive cerebellar ataxia type 1 (ARCA1), Emery-Dreifuss muscular dystrophy, and myogenic arthrogryposis, indicating its crucial role in neurological disorders [104–107]. The potential impact of Syne1’s circadian expression patterns on these disorders warrants further investigation, to elucidate the role of interaction between genetic predispositions and circadian gene regulation in their pathophysiology.

The increase in the HIPP circadian genes in substructures such as the centrosome and basal body raises the question of whether this reflects a compensatory response to maintain cellular homeostasis. Conversely, the decrease in BBSome circadian genes from 25 to 12.50% under epilepsy conditions suggests a potential decrease in cargo-targeting mechanisms. Ciliogenesis, elongation, and maintenance depend on the efficient functioning of the IFT system, with Kinesin-IFT-B being responsible for anterograde transport towards the ciliary tip, whereas dynein-IFT-A facilitates retrograde transport back to the base [108–110]. The shift from IFT-B circadian regulation to IFT-A may suggest altered intraciliary transport. This change may affect protein trafficking and cilia assembly, suggesting that epilepsy influences both the delivery and recycling of ciliary components.

In the SCN of mice on the HFD, we observed a 50% reduction in circadian cilia genes, with only four overlapping with the control group, and a notable shift in the peak timing of these genes from the dark to the light phase. There was particularly a notable reduction in the axoneme, ciliary basal body, centriole, and BBSome components, indicating that a high-fat diet may disrupt essential ciliary functions and signaling pathways. The complete absence of circadian genes in the intraciliary transport particle A and kinesin complex under HFD suggests a severe impairment in retrograde transport and motor protein function, which are crucial for ciliary maintenance and function. These findings highlight the potential impact of a high-fat diet on the circadian regulation of cilia genes in the SCN, potentially affecting the overall circadian rhythm and related physiological processes. Cilia length is known to be highly sensitive to nutrient type and quantity; HFD and obesity are associated with shorter cilia, while nutrient deprivation generally results in longer cilia [111–114], whereas nutrient deprivation leading to increased cilia length. Our findings are consistent with these observations, which suggest that dietary factors profoundly influence the circadian regulation of ciliary function and transport mechanisms [109, 110].

One limitation of our study is the potential variability introduced by using different mouse strains, which might impact the interpretation of our findings. We emphasize the need for future studies to directly compare these strains under similar conditions to further validate our results.

## Conclusion

Our study, though limited to six regions, provides initial insights into the region-specific circadian patterns of cilia-associated genes in the nocturnal mouse brain, demonstrating their complex and unique functional adaptations in each region. Our findings also reveal the adaptability of circadian cilia rhythms to physiological and pathological alterations, highlighting their sensitivity to environmental cues. Future research should focus on mapping circadian genes across a wider range of brain regions to gain a more comprehensive understanding of the role of cilia in brain functions.

## Electronic supplementary material

Below is the link to the electronic supplementary material.

**Supplementary Material 1:** List of cilia associated genes, CiliaCarta

**Supplementary Material 2:** Cilia gene transcripts that oscillate in a circadian manner in 6 mouse brain regions together with the oscillating signal’s period, lag, and amplitude

**Supplementary Material 3:** List of cilia associated genes found in each of the studied regions

**Supplementary Material 4:** Distribution of circadian cilia-associated genes across substructures in each studied brain region

## Data Availability

All circadian datasets and the BIO_CYCLE deep learning model are accessible on the CircadiOmics web portal (circadiomics.igb.uci.edu). Raw data for circadian cilia gene expressions can be found in Table S3.
